# Rare Presentation of a Posterior Mediastinal Cystic Schwannoma as a Large Pleural Effusion

**DOI:** 10.7759/cureus.1558

**Published:** 2017-08-10

**Authors:** Mujtaba Mubashir, Abdus Salam, Amyn Sonawalla, Huma Fatima, Saulat H Fatimi

**Affiliations:** 1 Medical School, The Aga Khan University; 2 Medical College, The Aga Khan University; 3 Department of Cardiothoracic Surgery, The Aga Khan University

**Keywords:** cystic schwannoma, schwannoma, pleural effusion, cystic, posterior mediastinal, thorax

## Abstract

A 46-year-old man presented with shortness of breath and recurrent, left-sided pleural effusions. A computed tomography (CT) scan of the chest showed a large, left-sided pleural effusion with a mass in the posterior mediastinum. A pleural tap showed lymphocytic exudate. The biopsy of the mass was inconclusive. A left posterolateral thoracotomy was performed, which showed a large posterior mediastinal mass adherent to the left lung. The mass was completely excised and the histopathology proved it to be giant predominantly cystic schwannoma. The pleural effusion resolved after tumor resection.

## Introduction

Schwannomas, also known as neurilemmomas, are rare, benign, neurogenic tumors of Schwann cells that form the outer insulating layer of peripheral nerves. In adults, neurogenic tumors account for around 20% of mediastinal tumors, with most of the schwannomas occurring in the posterior mediastinum [1]. Schwannomas are usually solid tumors, although cystic changes have been commonly described. Totally cystic schwannomas are much less commonly encountered and those occurring in the thorax, especially the mediastinum, are exceedingly rare; only 10 cases of predominantly cystic schwannomas have been reported in the literature [2] These tumors can be confused with other cystic masses of the mediastinum, both congenital and acquired [3]. Most mediastinal schwannomas are asymptomatic and may only be discovered on routine imaging [4]. We present a case of a 46-year-old man who presented with recurrent pleural effusions caused by a posterior mediastinal predominantly cystic schwannoma.

## Case presentation

A 46-year-old male, with no known comorbidity, presented with an episode of urticaria along with shortness of breath. According to the patient, the urticaria had started from the extremities, progressed to the trunk, and was associated with generalized itching and swelling of lips. This was accompanied by shortness of breath and a cough that progressed from dry to productive with white sputum. He had a history of undocumented weight loss over the last few months. The rest of the symptom review was unremarkable. The patient was a long-term smoker who had recently quit smoking in the past six months and was currently an active betel nut chewer. He had no known allergies, no prior hospitalizations or surgical procedures, and was not on any routine medications. His family history was unremarkable.

**Figure 1:**
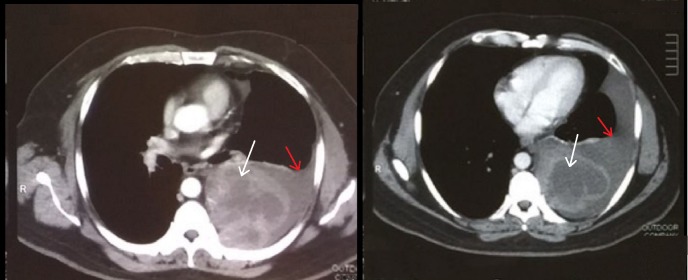
Preoperative axial CT scan of the thorax A large, left-sided posterior mediastinal mass is seen paravertebrally (white arrows) and is associated with a large, left-sided pleural effusion (red arrows). CT= computed tomography

A chest radiograph revealed a left-sided pleural effusion. A pleural tap showed a predominantly lymphocytic exudate. The pleural effusion was recurrent and had not settled despite repeated pleural taps. A subsequent computed tomography (CT) scan of the chest revealed a large, left-sided pleural effusion, as well as a large soft tissue mass in the posterior mediastinum in a paravertebral location without extension into the spinal canal, as can be seen in Figure 1.

**Figure 2:**
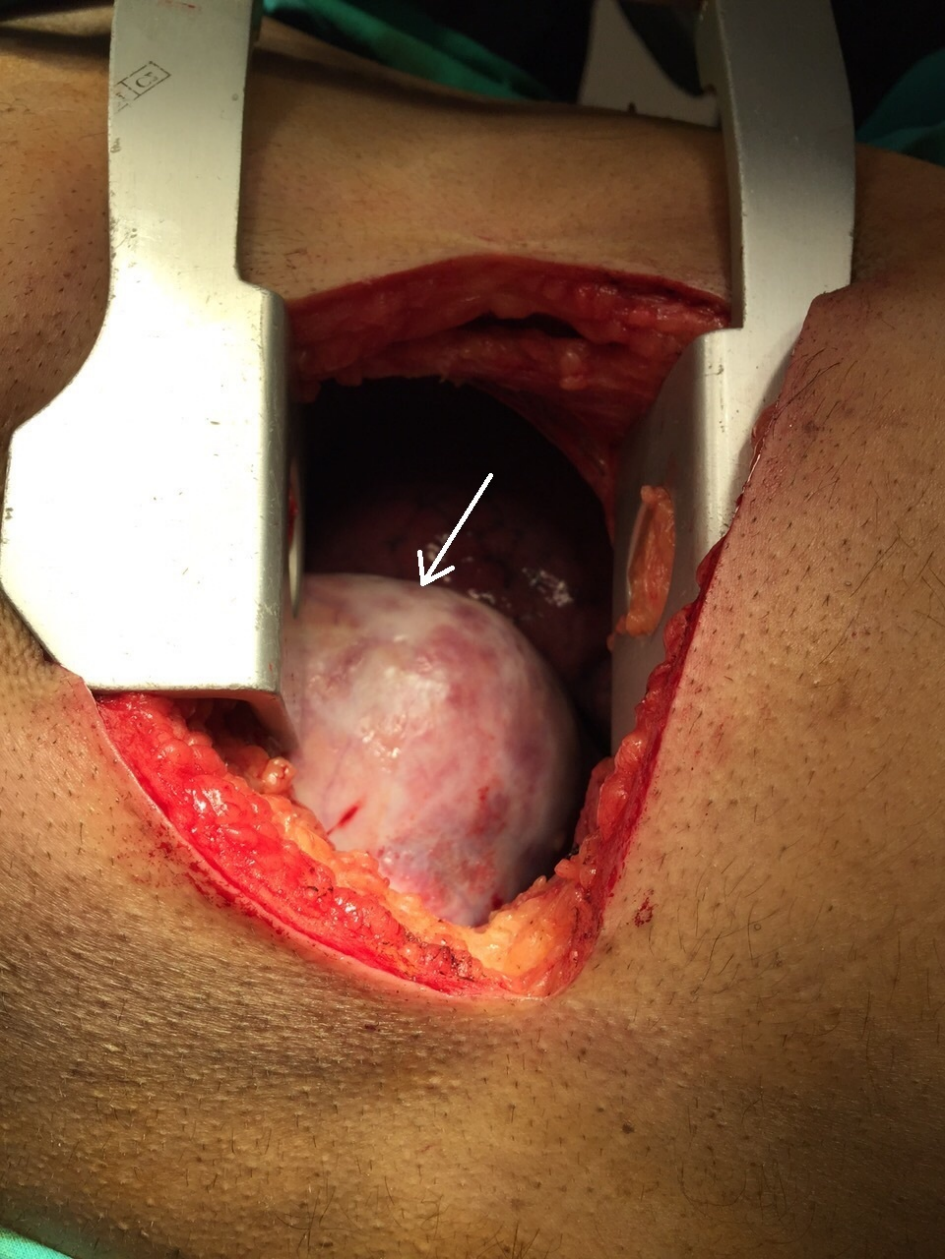
Intra-operative view of the mass (white arrow)

Biopsy attempts had proven inconclusive. Routine laboratory investigations were unremarkable. The patient was advised tumor excision. A left posterolateral thoracotomy was done, in which a large posterior mediastinal mass was seen adherent to the left lung, as seen in Figure 2.

**Figure 3:**
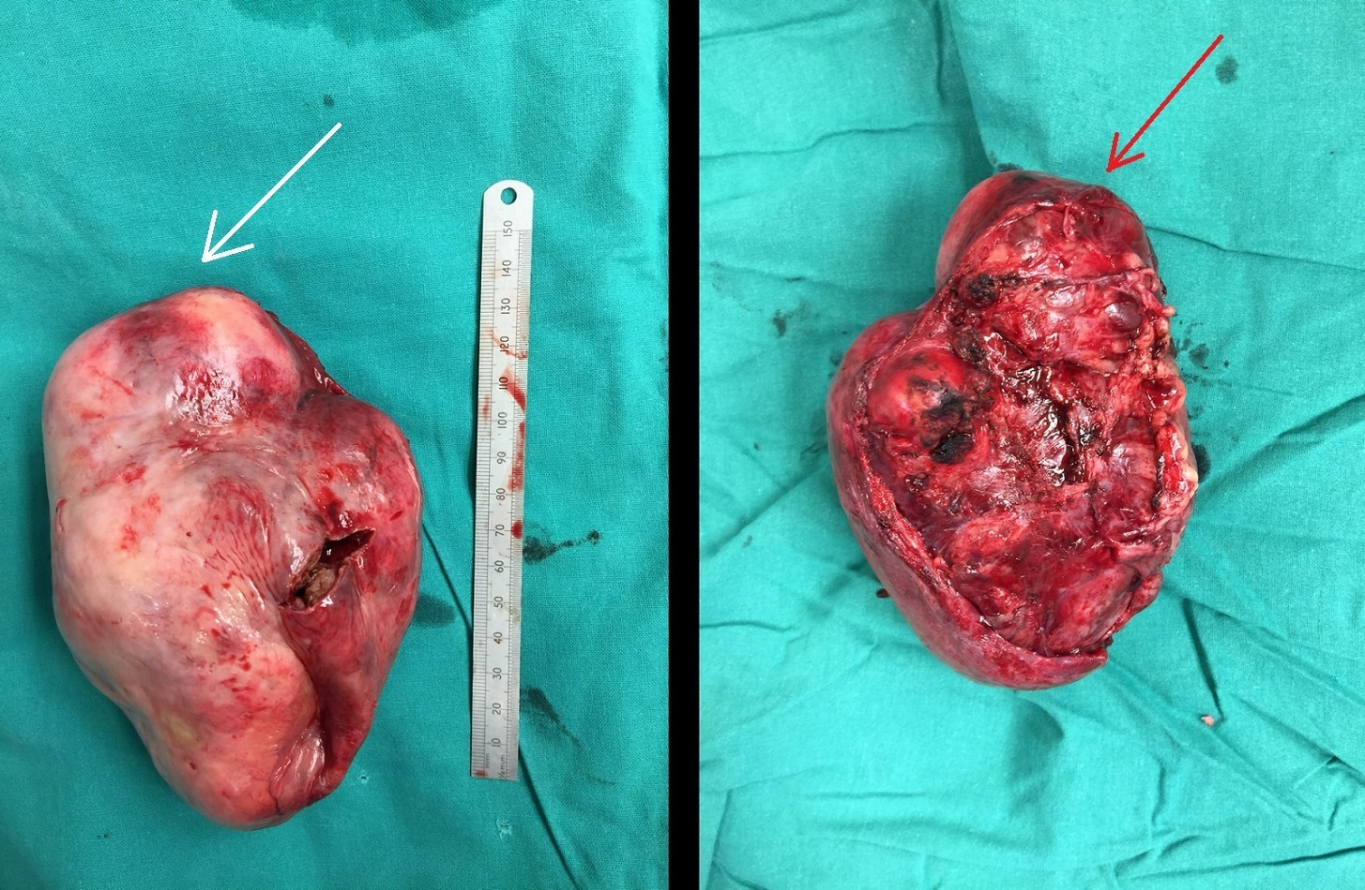
Excised mass This figure shows both surfaces of the excised mass, with the anterior surface shown on the left (white arrow) and the surface that was adherent to the lung shown on the right (red arrow). The mass measured 12.5 x 8.5 x 7.5 cm.

The mass was completely excised, as shown in Figure 3.

The patient had an uneventful postoperative recovery. Histopathology of the mass, which measured 12.5 x 8.5 x 7.5 cm, showed it to be a giant, predominantly cystic Schwannoma, with medium- to large-sized, congested hyalinized vessels with surrounding hemorrhage, as well as dense acute and chronic inflammatory changes without evidence of malignancy. The S-100 immunohistochemical stain was positive. Subsequent follow-up visits of the patient showed a complete resolution of the pleural effusion.

## Discussion

Posterior mediastinal masses may be classified as per their origin and morphology as neurogenic, esophageal, cystic, extramedullary hematopoiesis, or lymphomas. Of these, neurogenic tumors account for 80% of the posterior mediastinal masses. They may be peripheral, sympathetic, or paraganglionic in origin, and only a minority of these tumors are malignant (10% to 20%) [5]. In adults, the schwannoma is the most common posterior mediastinal neurogenic tumor.

Most schwannomas are asymptomatic and may be incidentally found on routine imaging. If symptoms do occur, they are usually because of their compressive effect on adjacent mediastinal structures, such as the airway, esophagus, heart, and great vessels. Rarely, when schwannomas are malignant, symptoms may also be due to the invasion of local structures and may present with respiratory symptoms, such as stridor, dyspnea, hemoptysis, and cough, or gastrointestinal (GI) symptoms such as dysphagia. Adjacent nerve involvement may present as hoarseness, pain, diaphragmatic paralysis, or Horner's syndrome. Hemothorax has also been reported with posterior mediastinal schwannomas [6].

Rarely, schwannomas may cause a pleural effusion. Very few cases have reported pleural effusions associated with schwannomas, and, in these reports, the schwannomas primarily originated from structures within the hemithorax such as from the intercostal nerves or from the chest wall [7]. In our case, the schwannoma was found to originate from the posterior mediastinum, unlike in existing literature. Pleural effusions associated with schwannomas are either reactionary to the presence of the tumor or hemorrhagic because of the rupture of the tumor [8]. The effusion in our case was most likely reactionary as evidenced by the resolution of the recurrent effusions after tumor resection.

Another unusual and noteworthy finding was the predominantly cystic nature of the schwannoma. Most schwannomas are solid and may have cystic components; however, totally cystic schwannomas are rare. Reported cases have usually been in close proximity to the spine and, consequently, have presented with neurological symptoms such as back ache and paresis [2], rather than the respiratory symptoms seen in our case.

Biopsy attempts may be inconclusive, as in our patient, and, as a result, surgical resection is necessary both for diagnosis and for treatment. Benign schwannomas have an excellent prognosis while malignant schwannomas may need additional radiotherapy [6].

## Conclusions

Giant cystic schwannomas may arise from the posterior mediastinum and cause recurrent pleural effusions. Patients may present with respiratory distress, and CT scans of the chest help identify the mass. Treatment involves a resection of the mass, after which the pleural effusions resolve spontaneously.
